# Integrative transcriptome and single-cell sequencing technology analysis of the potential therapeutic benefits of oleanolic acid in liver injury and liver cancer

**DOI:** 10.18632/aging.205349

**Published:** 2023-12-20

**Authors:** Hongji Xu, Qihang Yuan, Zhiqiang Wu, Yingsong Xu, Junhong Chen

**Affiliations:** 1 Department of Abdominal Surgery, Guiqian International General Hospital, Guiyang, Guizhou, China; 2 Department of General Surgery, First Affiliated Hospital of Dalian Medical University, Dalian, Liaoning, China; 3 Department of Thoracic Surgery, First Affiliated Hospital of Dalian Medical University, Dalian, Liaoning, China; 4 Department of Hepatobiliary and Pancreatic Surgery II, General Surgery Center, The First Hospital of Jilin University, Changchun, Jilin, China

**Keywords:** ferroptosis, oleanolic acid, HMOX1, liver cancer, treatment

## Abstract

Background: Oleanolic acid has important hepatoprotective effects and inhibits liver tissue carcinogenesis. The aim of this study was to investigate the mechanism of action of oleanolic acid in inhibiting liver injury and liver cancer.

Method: In this study, we applied differential gene analysis and gene enrichment analysis to identify the targets of oleanolic acid for the treatment of liver injury. And this study also applied Cibersort and GSVA methods to investigate the targets of oleanolic acid in liver injury. Based on oleanolic acid targets, we explored the major targets and further explored the role of the major targets in liver cancer. This study used the oncoPredict and the TIDE algorithm to predict the effect of oleanolic acid on drug resistance. Finally, the binding effect of oleanolic acid to relevant targets was explored using molecular docking techniques.

Result: In this study, oleanolic acid was found to inhibit liver injury and promote liver regeneration mainly by promoting elevated expression of HMOX1. Oleanolic acid can inhibit oxidative stress and promotes Ferroptosis in liver injury. In liver cancer, we identified that the main target of oleanolic acid is HMOX1 and HDAC1. And we determined that HMOX1 promotes Ferroptosis in liver cancer. This reduced the sensitivity of liver cancer to targeted therapies and immunotherapy. Molecular docking showed high binding of oleanolic acid to HDAC1 and HMOX1.

Conclusions: Oleanolic acid is an antioxidant by promoting high expression of HMOX1 and promotes the development of Ferroptosis in liver cancer and liver injury.

## INTRODUCTION

The liver is the largest digestive gland and detoxification organ in the body. Traditionally, liver injury refers to traumatic liver injury, which is damage to liver tissue and impaired liver function due to trauma. However, with the development of surgical techniques, there is a better system of treatment for traumatic liver injury in clinical practice.

Non-traumatic liver injury due to a variety of other hepatopathogenic factors has become a common type of liver injury in clinical practice. All types of liver injury can develop into liver cancer under certain carcinogenic factors. The common type of liver cancer is mainly liver cancer. Therefore, in this study, RNA-seq data from liver injury, cirrhosis, and liver cancer were analyzed to investigate the therapeutic mechanisms of related drugs.

Oleanolic acid is a pentacyclic triterpenoid that is a major component of herbs such as chasteberry and cyanophyll gall. Clinically oleanolic acid has a strong hepatoprotective effect, promoting liver regeneration and reducing the damaging effects of the liver [1]. It has been shown that drug nanocomplexes with oleanolic acid as the main component can improve the antioxidant capacity of the liver through the Nrf2 pathway and the anti-inflammatory capacity of the liver through the NF-kB pathway [2]. Oleanolic acid has a strong antioxidant effect, which is more related to the Nrf2 pathway. Oleanolic acid can promote the decrease of ROS in liver tissues by activating Nrf2 protein [3]. Relevant animal experiments have also shown that long-term oral administration of oleanolic acid can reduce the symptoms of inflammation and cholestasis in animal models of liver injury and has a strong hepatoprotective effect [4]. Some studies have also shown that oleanolic acid has strong anti-cholestatic effects, which may be related to the Nrf2 pathway and FXR pathway [5, 6].

In conclusion, oleanolic acid has good hepatoprotective effects and can inhibit the oxidative stress process and cholestasis of liver injury mainly through the Nrf2 pathway. Therefore, in this study, we analyzed the potential targets of oleanolic acid in liver cancer and liver injury to determine the potential mechanisms of oleanolic acid's anticancer effects.

## RESULTS

### Molecular characterisation of multiple types of liver injury

**Figure 1:**
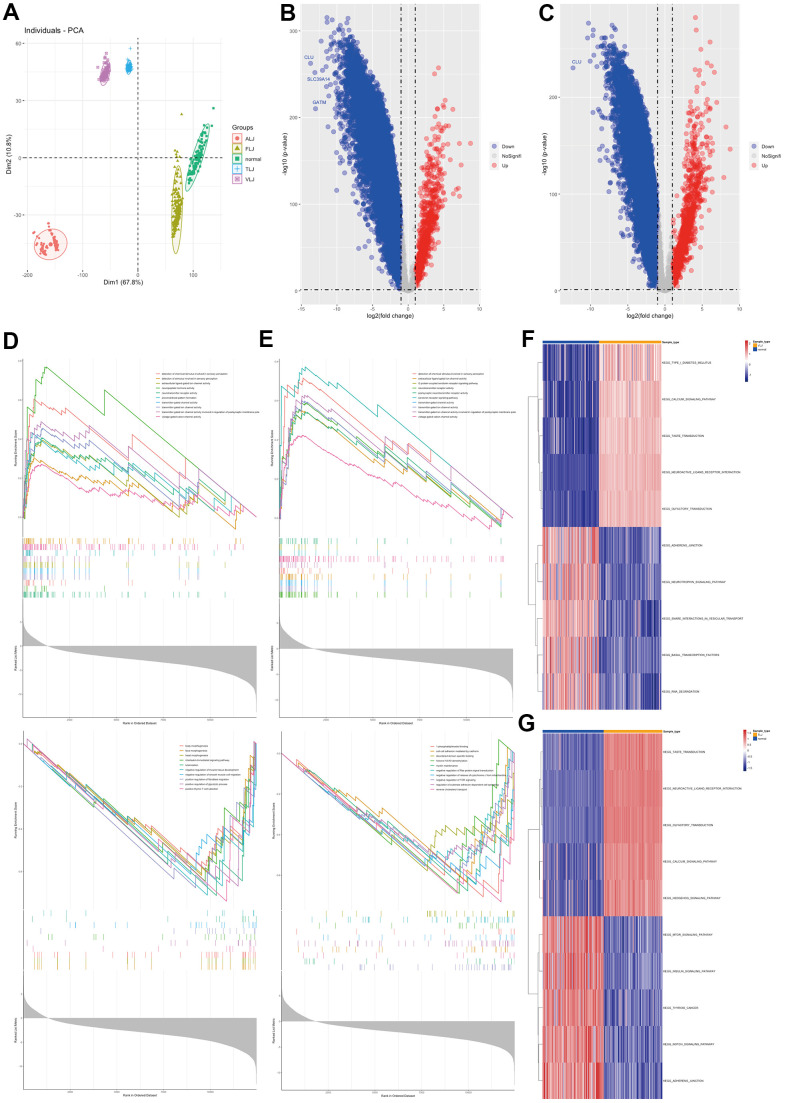
Molecular characterisation of VLJ and TLJ (**A**) PCA analysis of liver injury and normal. (**B**) Differential gene analysis of VLJ. (**C**) Differential gene analysis of TLJ. (**D**) GSEA analysis of VLJ based on GO gene set. (**E**) GSEA analysis of TLJ based on GO gene set. (**F**) GSVA analysis of VLJ based on KEGG pathway set. (**G**) GSVA analysis of TLJ based on KEGG pathway set.

In this study, RNA sequencing data from four types of liver injury tissues were collected from GEO database (Figure 1A). The differential gene analysis showed that the liver tissue of VLJ showed high expression of 842 genes and low expression of 10240 genes compared to normal tissue (Figure 1B). GSEA analysis showed that the elevated genes were mainly associated with ion transport channels and neuroreceptors, and those decreased genes were mainly associated with organ development and T cell selection (Figure 1D). Liver tissue from TLJ had high expression of 1170 genes and low expression of 9092 genes compared to normal liver tissue (Figure 1C). GSEA analysis showed that the genes that were elevated were similar to VLJ and those that were decreased were mainly associated with intercellular junctions and adhesion, particularly with the Ras and TOR pathways (Figure 1E). GSVA analysis showed elevated neurosensory-related pathways with reduced cell adhesion and transcription-related pathways in VLJ liver tissue (Figure 1F) and elevated neurosensory-related pathways with reduced cell adhesion and NOTCH pathways in TLJ liver tissue (Figure 1G).

**Figure 2:**
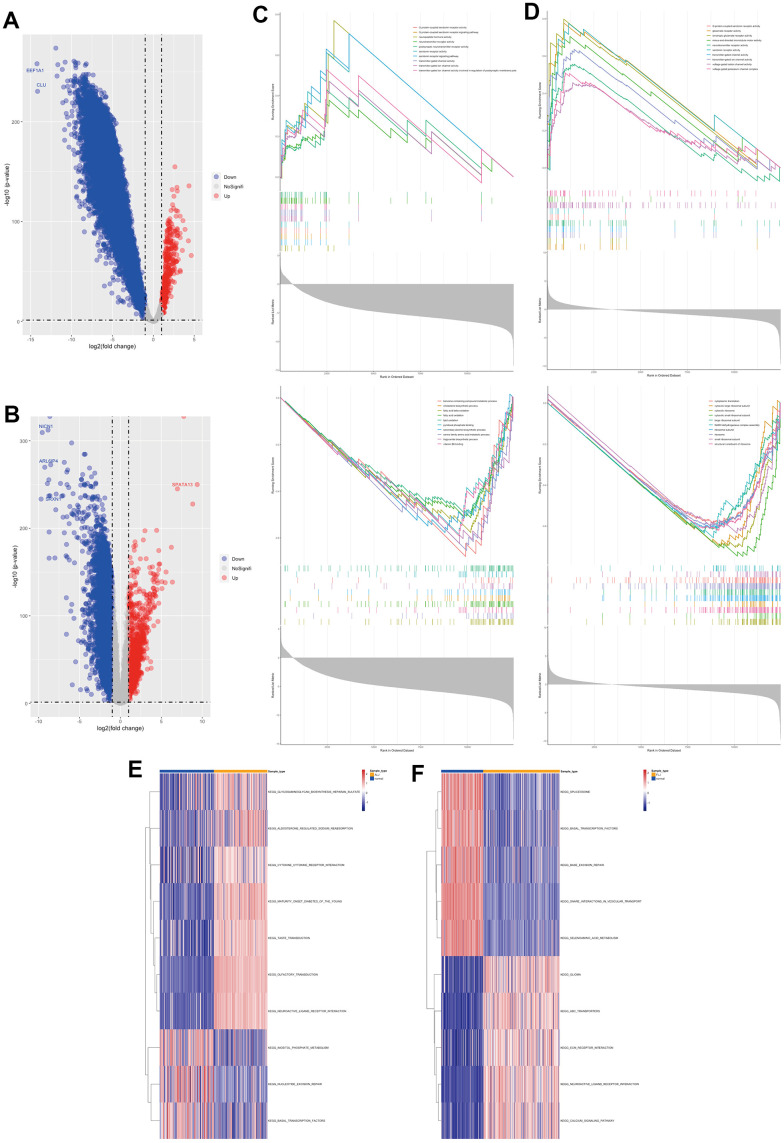
Molecular characterisation of ALJ and FLJ (**A**) Differential gene analysis of ALJ. (**B**) Differential gene analysis of FLJ. (**C**) GSEA analysis of ALJ based on GO gene set. (**D**) GSEA analysis of FLJ based on GO gene set. (**E**) GSVA analysis of ALJ based on KEGG pathway set. (**F**) GSVA analysis of FLJ based on KEGG pathway set.

This study also analysed the gene profile characteristics of ALJ and FLJ. The differential gene analysis showed that there were 351 highly expressed genes and 11419 lowly expressed genes in ALJ compared to normal tissue (Figure 2A). GSEA analysis showed that the genes that were elevated were also mainly associated with ion transport channels and neuroreceptors, and the genes that were decreased were mainly associated with alcohol and fat metabolism (Figure 2C). There were 949 highly expressed genes and 4706 lowly expressed genes in FLJ compared to normal tissue (Figure 2B). GSEA analysis showed that the genes that were elevated were also mainly associated with ion transport channels and neuroreceptors, and those that were decreased were mainly associated with ribosomes (Figure 2D). GSVA analysis also showed elevated neurosensory-related pathways and reduced transcription-related pathways in ALJ liver tissue (Figure 2E) and elevated neurosensory-related pathways and reduced transcription-related pathways in FLJ liver tissue (Figure 2F). All of these suggest that there are more similarities in the genetic profiles of the four types of liver injury.

### Common characteristics of liver injury and targets of oleanolic acid

**Figure 3:**
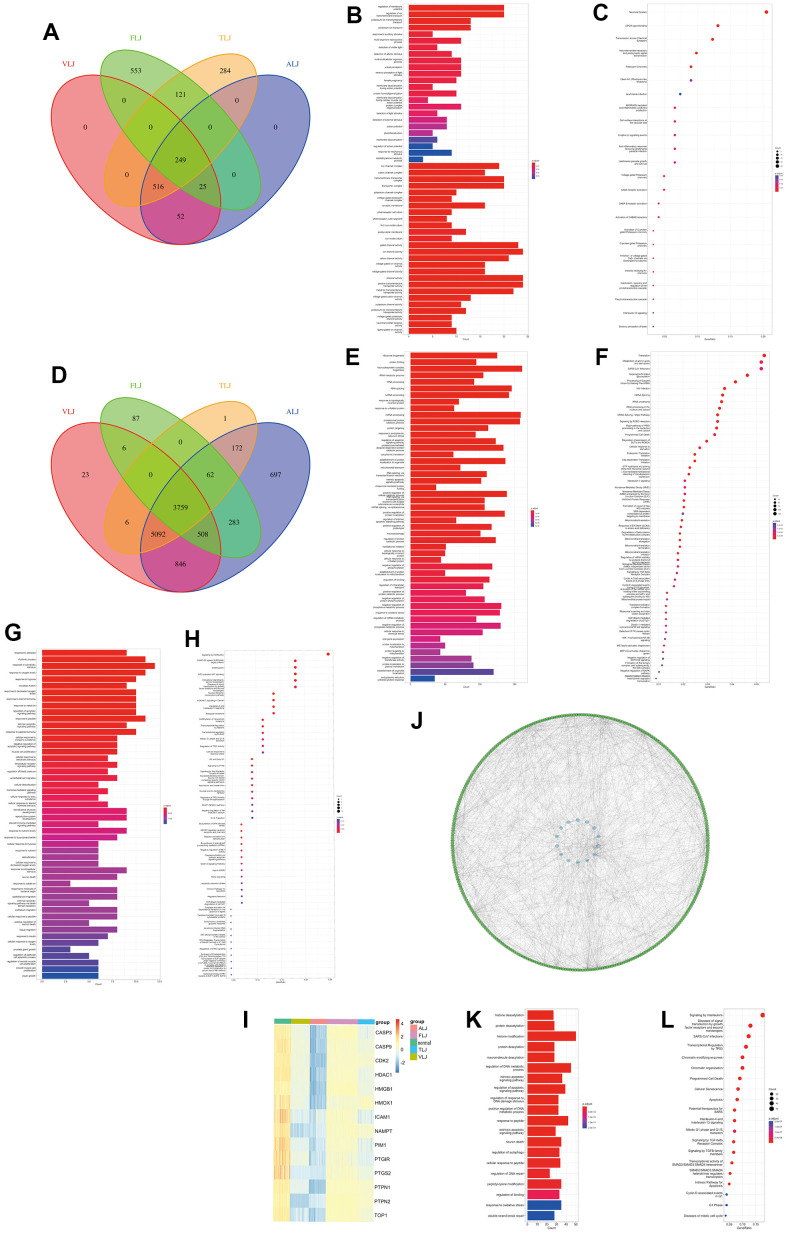
Common characteristics of liver injury and targets of oleanolic acid (**A**) Highly expressed genes of the liver injury. (**B**) Gene function of highly expressed genes. (**C**) Cellular pathways of highly expressed genes. (**D**) Lowly expressed genes of the liver injury. (**E**) Gene function of lowly expressed genes. (**F**) Cellular pathways of lowly expressed genes. (**G**) Gene function of oleanolic acid targets. (**H**) Cellular pathways of oleanolic acid targets. (**I**) Expression of 11 liver injury-related oleanolic acid targets. (**J**) PPI analysis of 11 liver injury-related oleanolic acid targets. (**K**) Gene function of genes in PPI analysis. (**L**) Cellular pathways of genes in PPI analysis.

In this study, we intersected the differential genes of the four liver injuries and we found 249 highly expressed genes (Figure 3A) and 3759 lowly expressed genes (Figure 3D) in the four liver injuries. In terms of gene function, most of the highly expressed genes were associated with ion channels and extracellular stimulation (Figure 3B), whereas most of the lowly expressed genes were associated with intracellular transcriptional translation (Figure 3E). In terms of cellular pathways, most of the highly expressed genes were associated with G protein receptors and neuroreceptor pathways (Figure 3C), while most of the lowly expressed genes were associated with transcriptional-translational pathways (Figure 3F).

Oleanolic acid, an important small molecule in Chinese medicine, was found to have 44 targets (Supplementary Table 1) according to the database, which were mainly related to oxidative stress and cell growth in terms of gene function (Figure 3G). In terms of cellular pathways, these targets are related to the interleukin pathway, AKT pathway, p53 pathway and Nrf2 pathway (Figure 3H).

### Key targets and protein networks of oleanolic acid in liver injury

In this study, 11 liver injury-related oleanolic acid targets were obtained from 3759 low-expressed liver injury genes and 44 oleanolic acid-related targets (Figure 3I), and all of these genes were lowly expressed in liver injury. By PPI analysis we identified 328 genes that were lowly expressed in liver injury associated with 14 targets (Figure 3J). In terms of gene function, these genes were mainly associated with DNA repair, protein acetylation, and oxidative stress (Figure 3K). In terms of cellular pathways, these genes are mainly associated with the interleukin pathway, the P53 pathway and the cell cycle pathway (Figure 3L).

**Figure 4:**
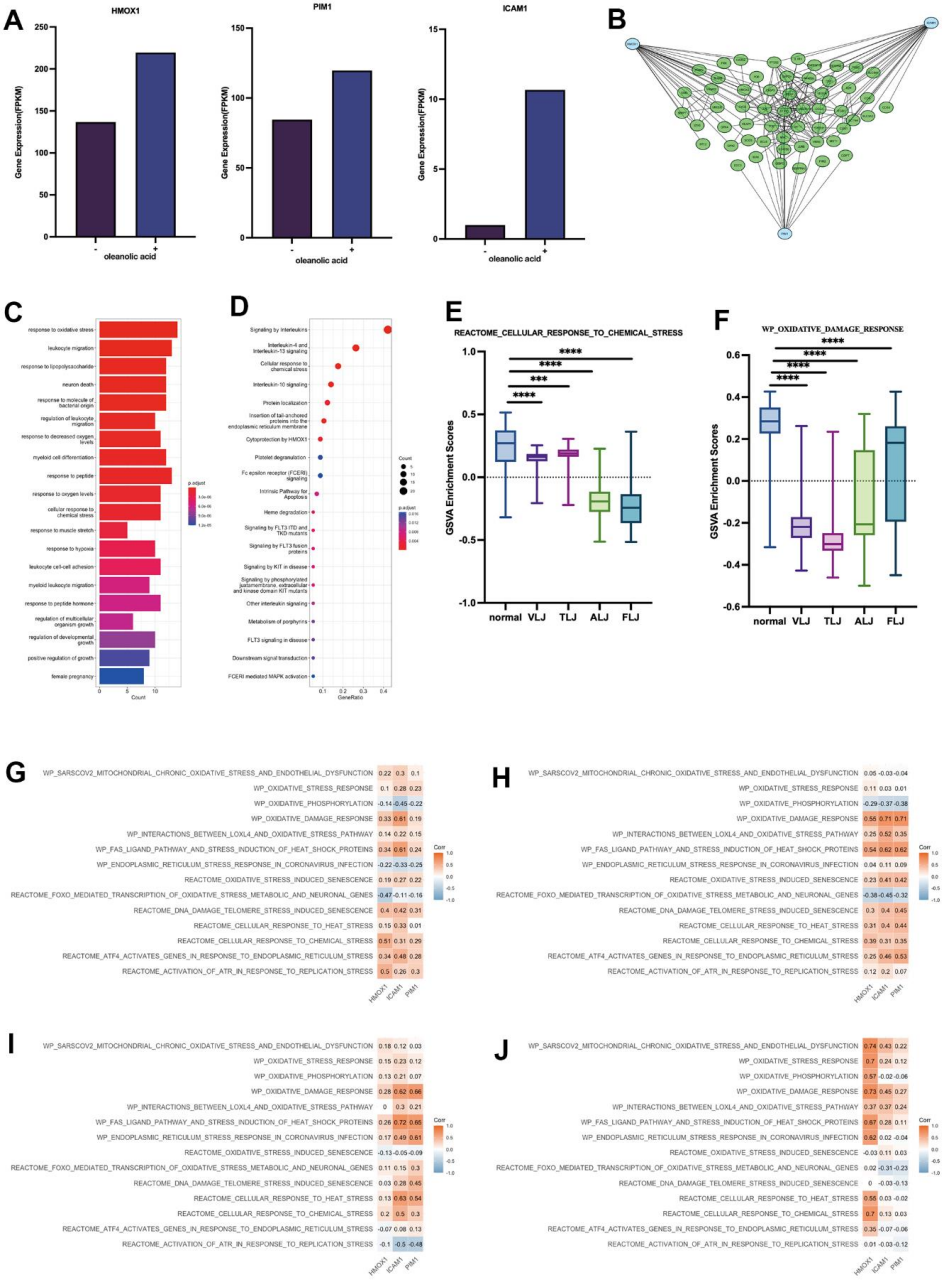
Key targets and protein networks of oleanolic acid (**A**) High expression of HMOX1, PIM1 and ICAM1 through oleanolic acid-related expression profiles. (**B**) PPI analysis of 3 key targets of oleanolic acid. (**C**) Gene function of genes in PPI analysis. (**D**) Cellular pathways of genes in PPI analysis. (**E**) GSVA analysis of response to chemical stress in liver injury. (**F**) GSVA analysis of response to oxidative damage in liver injury. (**G**) Relationship between key targets of oleanolic acid and 14 oxidative stress pathways in VLJ. (**H**) Relationship between key targets of oleanolic acid and 14 oxidative stress pathways in TLJ. (**I**) Relationship between key targets of oleanolic acid and 14 oxidative stress pathways in ALJ. (**J**) Relationship between key targets of oleanolic acid and 14 oxidative stress pathways in FLJ.

In this study, we obtained that oleanolic acid promotes high expression of HMOX1, PIM1 and ICAM1 in hepatocellular carcinoma cells through oleanolic acid-related expression profiles, which further suggests that HMOX1, PIM1 and ICAM1 are key targets of oleanolic acid (Figure 4A). The protein network associated with the three genes was further explored, with the three genes associated with 55 genes which decreased expression in liver-injured tissues (Figure 4B). In terms of gene function, these genes are mainly associated with oxidative stress (Figure 4C). In terms of cellular pathways, these genes are mainly associated with mutations in the interleukin pathway and in the FLT3 and KIT genes (which are also mainly associated with leukaemia) (Figure 4D). GSVA analysis revealed a lower response to chemical stress (Figure 4E) and oxidative damage (Figure 4F) in the four liver-injured tissues, resulting in a lower resistance to oxidative stress in the liver-injured tissues compared to the normal liver tissues. The study also analysed the relationship between the key targets of oleanolic acid and 14 oxidative stress pathways in four types of liver-injured tissues. The results showed that most of the oxidative stress pathways were positively correlated with the main targets of oleanolic acid in four types of liver injury, including VLJ (Figure 4G), TLJ (Figure 4H), ALJ (Figure 4I) and FLJ (Figure 4J). This suggests that oleanolic acid inhibits liver injury and promotes the regeneration process of liver tissue mainly through the antioxidant and immune pathways. Meanwhile, HMOX1, an important antioxidant stress enzyme, and PIM1, an important proto-oncogene, can promote the differentiation of fibrous tissue into liver tissue. Both of these suggest that oleanolic acid has good antioxidant and pro-hepatic regenerative functions.

### Immunological and ferroptosis characteristics of liver injury

**Figure 5:**
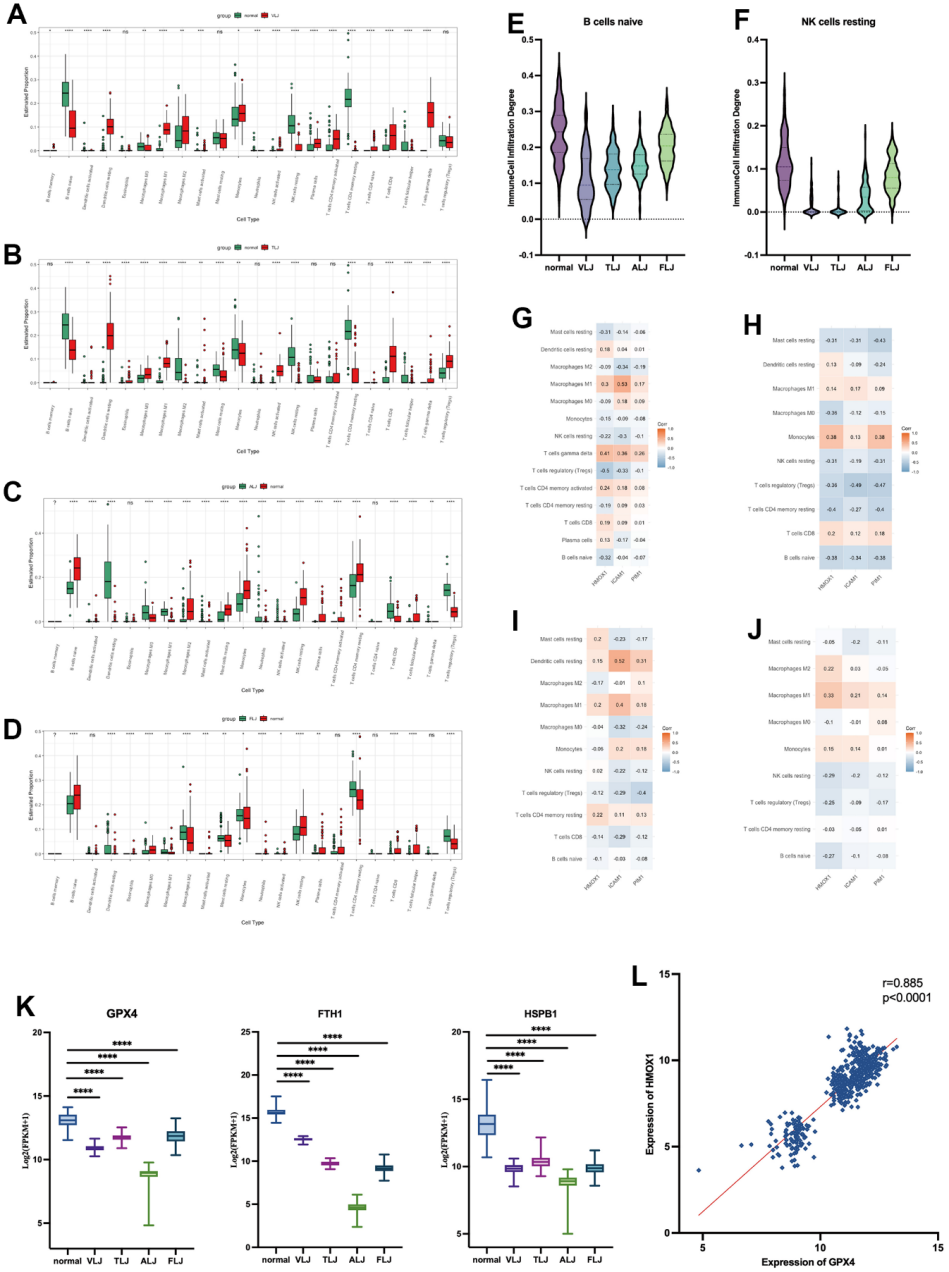
Immunological and Ferroptosis characteristics of liver injury (**A**) immune infiltration analysis of VLJ. (**B**) immune infiltration analysis of TLJ. (**C**) immune infiltration analysis of ALJ. (**D**) immune infiltration analysis of FLJ. (**E**) Decrease in naive B cells of liver injury. (**F**) Decrease in resting NK cells of liver injury. (**G**) Correlation between the key targets and the degree of immune cell in VLJ. (**H**) Correlation between the key targets and the degree of immune cell in TLJ. (**I**) Correlation between the key targets and the degree of immune cell in ALJ. (**J**) Correlation between the key targets and the degree of immune cell in FLJ. (**K**) Expression of GPX4, FTH1 and HSPB1 in liver injury. (**L**) Correlation between expression of GPX4 and HMOX1.

In this study, immune infiltration analysis of different liver injury tissues was performed using the Cibersort algorithm. It was found that there were more infiltrations of CD8 positive T cells and dormant DC cells in VLJ and TLJ liver tissues (Figure 5A, 5B). In addition, Treg cells and dormant DC cells were present in ALJ and FLJ liver tissues (Figure 5C, 5D). This suggests partial activation of immune function in VLJ and TLJ and suppression of immune function in ALJ and FLJ. However, there was a decrease in naive B cells (Figure 5E) and resting NK cells (Figure 5F) in all four types of liver injury, suggesting that the nature of liver injury still leaves liver tissue with reduced immune function, which makes the liver more susceptible to carcinogenesis. We also investigated the correlation between the oleanolic acid key targets and the degree of partial immune cell (immune cell infiltration >0 in half of cases) infiltration in various liver-injured tissues. In VLJ liver tissue, the key targets of oleanolic acid were positively correlated with M1 macrophages and negatively correlated with Tregs cells (Figure 5G). In TLJ liver tissue, the key targets of oleanolic acid were positively correlated with monocytes and negatively correlated with Tregs (Figure 5H). In ALJ liver tissue, the key targets of oleanolic acid were positively correlated with dormant DC cells and negatively correlated with Tregs cells (Figure 5I). In FLJ liver tissue, the key targets of oleanolic acid were positively correlated with M1 macrophages and negatively correlated with Tregs cells and dormant NK cells (Figure 5J). In conclusion, we suggest that oleanolic acid can restore liver immune function and prevent liver cancer by increasing the expression of the primary target and inhibiting the infiltration of Tregs cells.

In this study, low expression of Ferroptosis suppressor genes such as GPX4, FTH1 and HSPB1 was also found in all four types of liver injury tissues (Figure 5K). The increased expression of HMOX1 protein was accompanied by a high expression of GPX4 protein in all liver injury tissues (Figure 5L). This suggests that iron death proceeds in a variety of liver injuries and that oleanolic acid may inhibit the development of Ferroptosis by increasing HMOX1.

### The role of oleanolic acid targets in cancer

**Figure 6:**
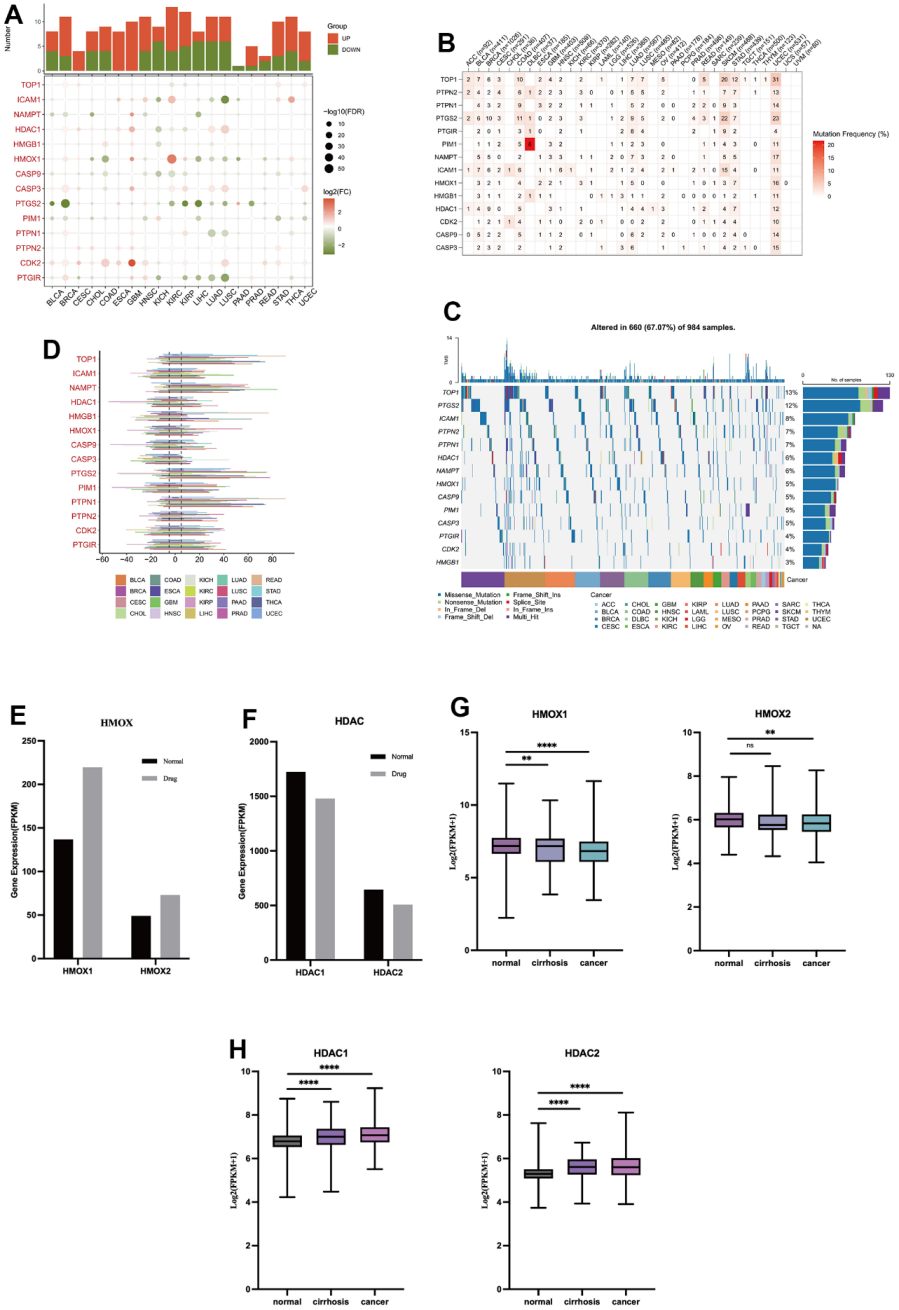
The role of oleanolic acid targets in cancer (**A**) Expression of oleanolic acid targets in pan-cancer. (**B**) Mutation frequency of oleanolic acid targets in pan-cancer. (**C**) Mutation types of oleanolic acid targets in pan-cancer. (**D**) Copy number mutations of oleanolic acid targets in pan-cancer. (**E**) Expression of HMOX1 and HMOX2 in liver cancer with oleanolic acid. (**F**) Expression of HDAC1 and HDAC2 in liver cancer with oleanolic acid. (**G**) Expression of HMOX1 and HMOX2 in liver cancer. (**H**) Expression of HDAC1 and HDAC2 in liver cancer.

This study uses pan-cancer data from TCGA to explore the role of liver injury-related oleanolic acid targets in 20 cancer types. Fourteen liver-damaging oleanolic acid targets were generally lowly expressed in cancer and HMOX1 was lowly expressed in liver cancer (Figure 6A). The 14 liver injury-associated oleanolic acid targets also had low somatic mutation rates in cancer, and HMOX1 had a 1% somatic mutation rate in liver cancer (Figure 6B) compared to a 5% mutation rate in cancer as a whole (Figure 6C). In terms of copy number mutations, 14 oleanolic acid targets showed some copy number increase in cancer, but the extent of amplification was not significant (Figure 6D). Using oleanolic acid-related liver cancer sequencing data, we found that oleanolic acid decreased the expression of HDAC1 and HDAC2 (Figure 6F) and increased the expression of HMOX1 and HMOX2 (Figure 6E). Using sequencing data related to liver cancer and cirrhosis, we found that HDAC1 and HDAC2 (Figure 6H) expression was elevated in liver cancer and cirrhosis, and HMOX1 and HMOX2 expression was decreased (Figure 6G). These suggest that HMOX1 may inhibit the progression of liver cancer while HDAC1 may promote tumor development, and oleanolic acid may act as an anticancer agent through the above targets.

### Role of HDAC1 and HMOX1 in liver cancer

**Figure 7:**
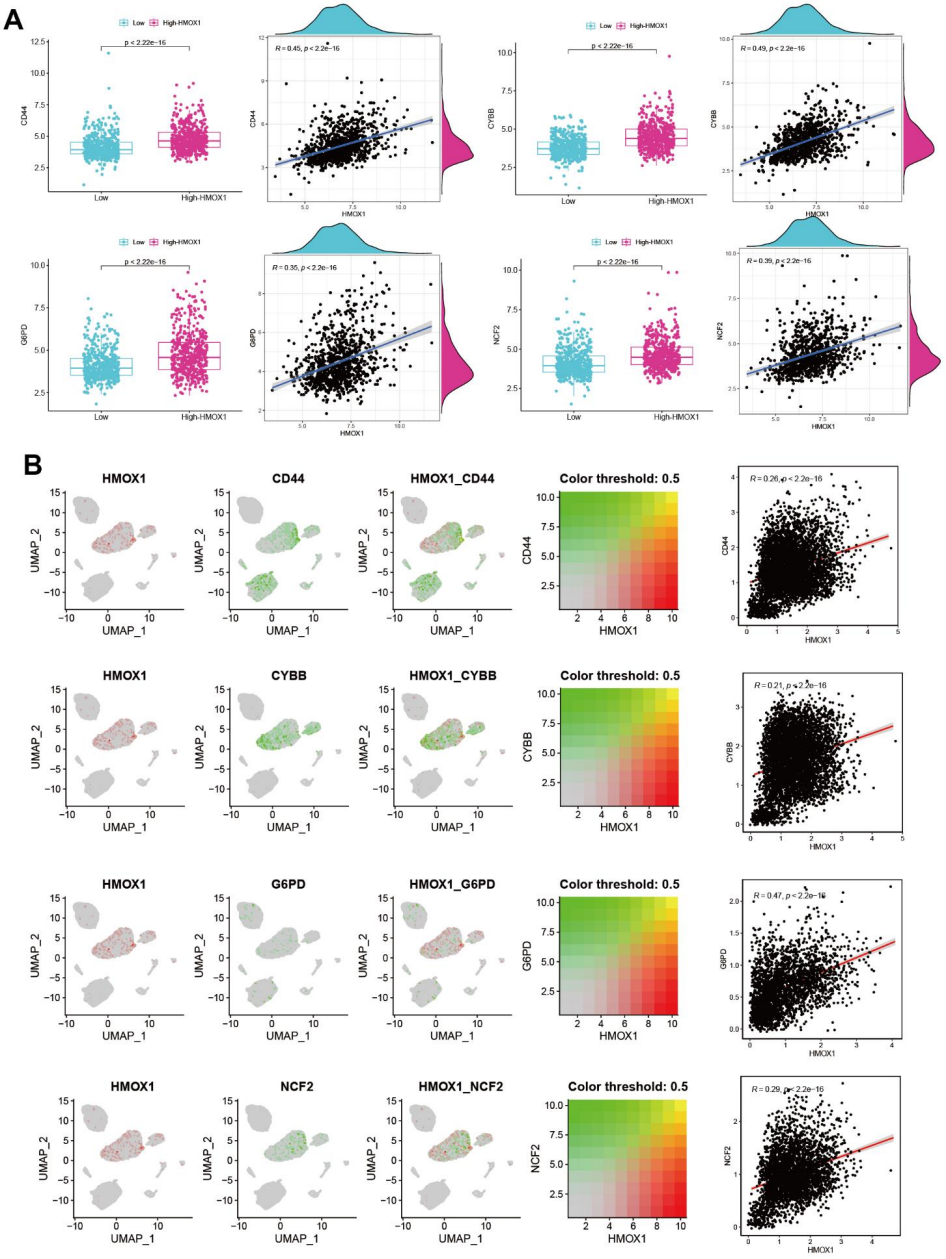
HMOX1 and Ferroptosis in liver cancer (**A**) Ferroptosis promoting genes were strongly correlated with HMOX1 based on RNA-seq. (**B**) Ferroptosis promoting genes were strongly correlated with HMOX1 in liver cancer microenvironment.

Based on RNA-seq data from 968 liver cancer-related patients, we explored the effect of the HMOX1 gene on iron death-related genes. The findings revealed that the Ferroptosis promoting genes CD44, CYBB, G6PD, and NCF2 were all strongly correlated with HMOX1. And HMOX1 could promote the expression of the above genes, thus promoting Ferroptosis (Figure 7A). Also based on scRNA-seq data, we have a large expression correlation between HMOX1 in the liver cancer microenvironment and the above-mentioned genes promoting Ferroptosis in the same cell populations (Figure 7B). This all suggests that HMOX1 can promote high expression of Ferroptosis promoting genes and promotes Ferroptosis of liver cancer.

**Figure 8:**
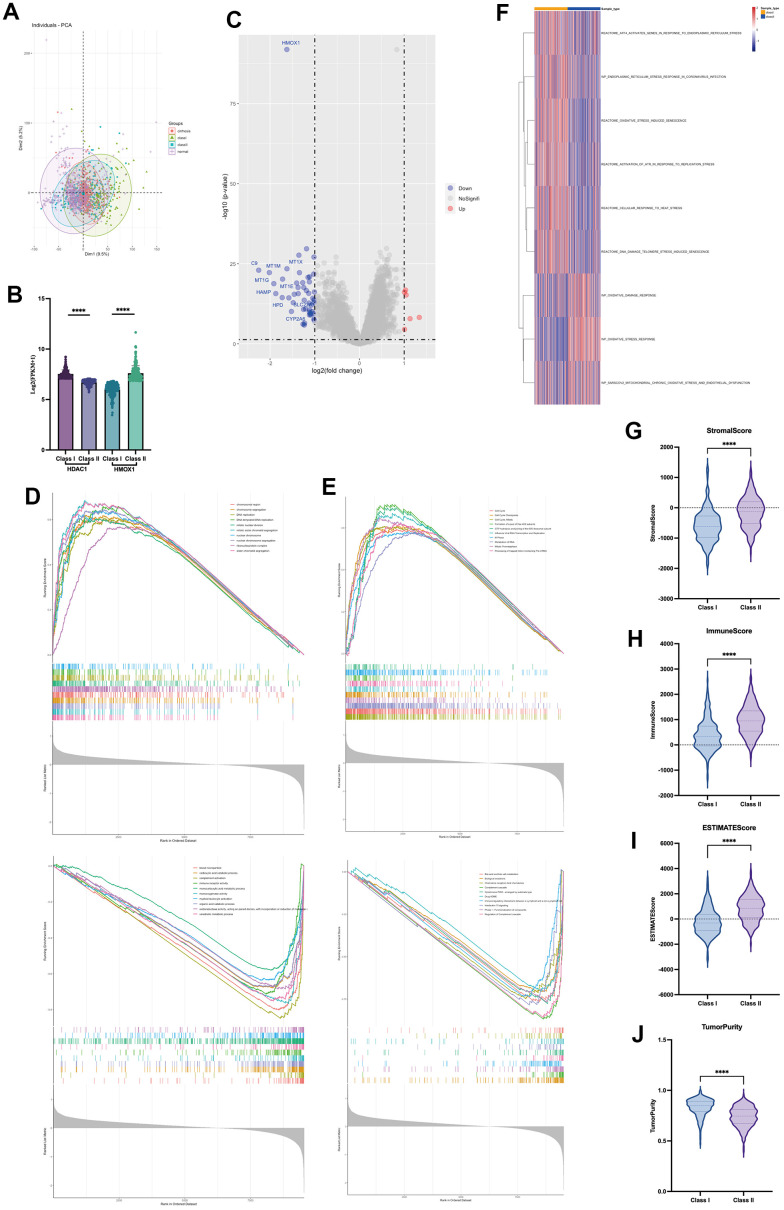
Role of HDAC1 and HMOX1 in liver cancer (**A**) PCA analysis of two groups. (**B**) Expression of HDAC1 and HMOX1 of two groups. (**C**) Volcano of two groups. (**D**) GSEA analysis of differential genes based on GO. (**E**) GSEA analysis of differential genes based on ReactomePA. (**F**) GSVA analysis of two groups based on oxidative stress pathway. (**G**) Stromal score in two groups. (**H**) Immune score in two groups. (**I**) Estimate score in two groups. (**J**) Tumour purity in two groups.

**Figure 9:**
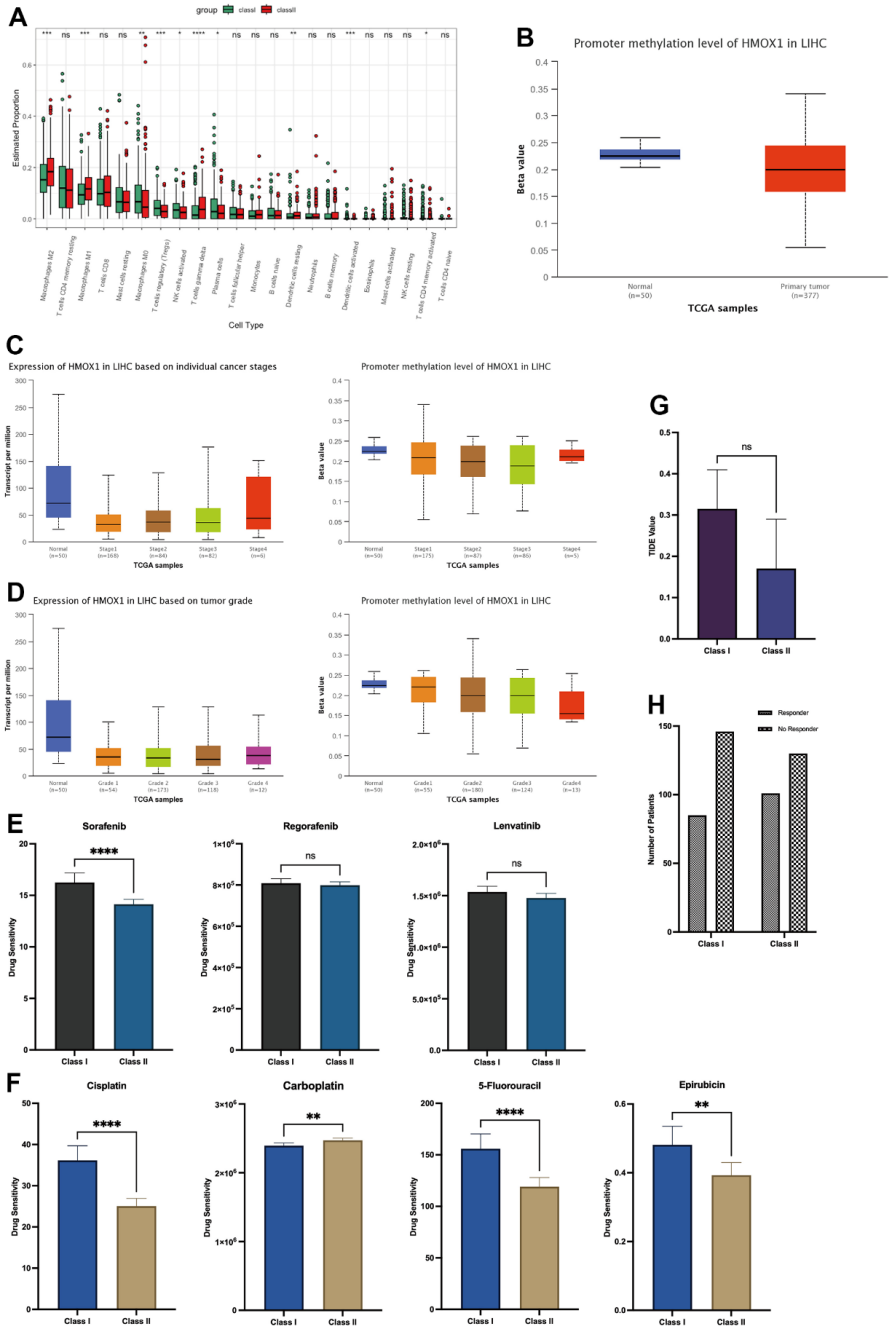
Microenvironmental effects HDAC1 and HMOX1 in liver cancer (**A**) Immunocytic infiltration analysis of two groups. (**B**) Methylation of HMOX1 in liver cancer. (**C**) Expression and Methylation of HMOX1 in clinical stage. (**D**) Expression and Methylation of HMOX1 in pathology grade. (**E**) Drug sensitivity analysis of Sorafenib, Regorafenib and Lenvatinib based on Oncopredict. (**F**) Drug sensitivity analysis of Cisplatin, Carboplatin, 5-Fluorouracil and Epirubicin based on Oncopredict. (**G**) TIDE Value in two groups. (**H**) Response to immunotherapy in two groups.

This study utilized two groups of significantly different liver cancer samples for analysis, with 231 patients in each group (Figure 8A). The difference between the two groups of patients was that the Class I group had higher expression of HDAC1 and lower expression of HMOX1, while the opposite was true for the Class II group (Figure 8B). Differential gene analysis showed the presence of 48 low and 6 high expressed genes in Class I group compared to Class II group (Figure 8C). Oxidative stress-related GSVA pathway analysis showed that Class II had better antioxidant capacity (Figure 8F). In terms of gene function, GSEA analysis showed that the Class I group had a higher DNA replication capacity with a lower immune response than the Class II group (Figure 8D). In terms of pathway activation, GSEA analysis showed that the Class I group had higher cell cycle activity and lower immune response and drug response than the Class II group (Figure 8E). Estimate analysis showed that the Class I group had lower stromal score (Figure 8G), immune score (Figure 8H), and Estimate score (Figure 8I), and higher tumor purity (Figure 8J) than the Class II group, suggesting a higher malignancy of liver cancer in the Class I group. Cibersort immune cell analysis showed a higher degree of infiltration of Tregs cells in Class I, but a higher degree of infiltration of M1 macrophages, M2 macrophages, and activated DC cells in the Class II group (Figure 9A), suggesting a more pronounced microenvironmental immunosuppression in Class I. Using the ULCAN database, we found significant elevated methylation of HMOX1 in liver cancer (Figure 9B), and we found the expression of HMOX1 with the degree of methylation in different clinical stages (Figure 9C) and different pathological stages (Figure 9D). This suggests that the regulation of HMOX1 may be dominated by gene methylation. Drug sensitivity analysis showed a higher sensitivity of Class II to sorafenib in terms of targeted drugs (lower IC50 values) (Figure 9E). Drug sensitivity analysis showed that the Class II group had higher sensitivity (lower IC50 values) to cisplatin, 5-fluorouracil and epirubicin, while the Class I group had higher sensitivity to carboplatin in terms of chemotherapeutic agents (Figure 9F). TIDE immunotherapy sensitivity analysis showed that the Class group had higher TIDE values (Figure 9G). This demonstrates that the Class I group has a lower sensitivity to immunotherapy and has a lower number of patients who respond to immunotherapy (Figure 9H). All of the above indicate that high expression of HMOX1 and low expression of HDAC1 can inhibit the progression of liver cancer and promote the pharmacological treatment of liver cancer, which provides the basis for the clinical application of oleanolic acid.

### Molecular docking and experimental validation

**Figure 10:**
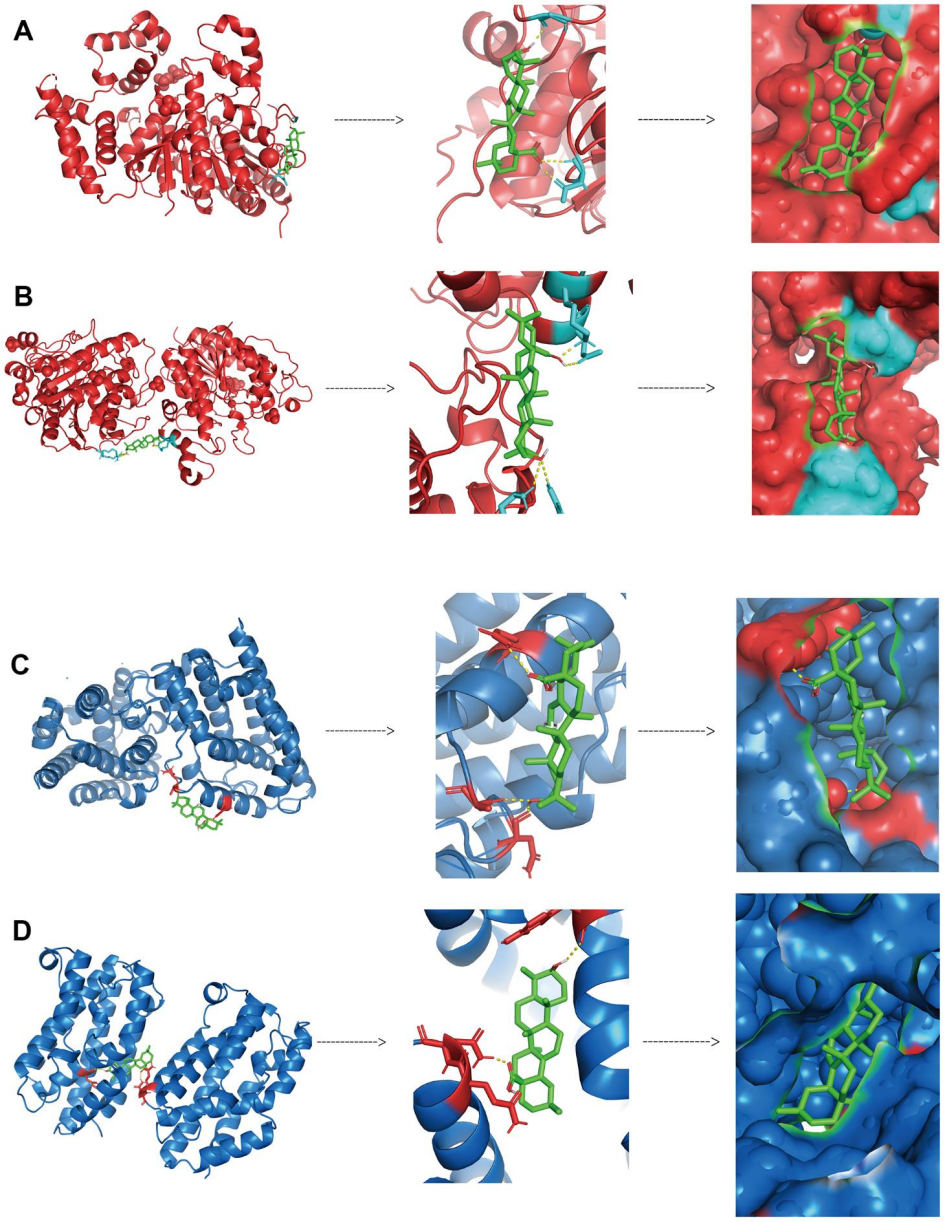
Binding between oleanolic acid and key targets (**A**) The docking binding of HDAC1 protein and oleanolic acid. (**B**) The docking binding of HDAC2 protein and oleanolic acid. (**C**) The docking binding of HMOX1 protein and oleanolic acid. (**D**) The docking binding of HMOX2 protein and oleanolic acid.

We further validated the binding of HDAC1, HDAC2, HMOX1 and HMOX2 proteins to oleanolic acid by analyzing the docking technique. The docking binding energy of HDAC1 and HDAC2 protein to oleanolic acid was 7.99kcal/mol (Figure 10A) and 7.32kcal/mol (Figure 10B). The docking binding energy of HMOX1 and HMOX2 protein to oleanolic acid was 6.46kcal/mol (Figure 10C) and 7.89kcal/mol (Figure 10D). This suggest that HDAC1 and HMOX1 are important targets of oleanolic acid for liver cancer inhibition.

## DISCUSSION

The liver is the body's largest digestive gland and an important detoxification organ, as almost all foreign substances are metabolised and broken down by the liver. Most clinical liver injuries are caused by the destruction of foreign substances in the liver. The main causes of liver damage are hepatitis viruses, alcohol, excessive fat accumulation and liver grafts. The accumulation of these foreign substances can lead to an increase in ROS in liver tissue and activation of the immune response, leading to further liver damage and ultimately to fibrosis and loss of liver function.

Oleanolic acid is a clinically important hepatoprotective agent and is a common small molecule found in a variety of herbal medicines. Numerous studies have found oleanolic acid to have good antioxidant effects in many diseases. In common brain tissue injury, oleanolic acid can activate the expression of antioxidant stress enzymes through the Nrf2/HOMX1 pathway, thereby down-regulating ROS in injured brain tissue in subarachnoid hemorrhage and achieving the function of protecting brain tissue [7]. In diabetes, oleanolic acid can reduce cellular oxygen stress through agonism of the Akt and MAPK pathways, thereby inhibiting the effects of insulin resistance during diabetes treatment [8]. In Alzheimer's disease, oleanolic acid regulates UCP2 expression via STC-1 to attenuate oxidative stress and β-amyloid levels in N2a/APP695swe cells [9]. It has also been shown that skin damage caused by airborne PM2.5 particles can be alleviated by the antioxidant effect of oleanolic acid [10]. In hypertension, hypertension promotes endoplasmic reticulum stress and ROS accumulation in kidney cells, while oleanolic acid inhibits the progression of hypertensive nephropathy by reducing ROS levels [11]. Similarly in diabetic patients, there is enhanced oxidative stress in the vascular endothelium, and OA protects endothelial cells from oxidative stress-induced apoptosis, which is associated with the AKT/eNOS signaling pathway [12]. In summary, we can see that oleanolic acid has a good anti-oxidative stress ability and can inhibit the apoptotic process of tissue cells under oxidative stress conditions. In addition, oleanolic acid also has anti-fibrotic effects, which are also more related to the effects of oxidative stress. In pulmonary fibrosis, oleanolic acid modulates the AKT/NF-κB pathway to reduce cytokines to further inhibit the progression of pulmonary fibrosis [13]. Similarly in liver fibrosis, nanoparticles of oleanolic acid protect hepatocytes and inhibit the fibrotic process of hepatocytes by reducing TGF-β1 levels and oxidative stress in PM2.5 exposed hepatocytes [14]. Oleanolic acid, an important antioxidant drug, also has some anticancer effects. In hepatocellular carcinoma, oleanolic acid inhibited the increase of ROS, thus increasing the sensitivity of hepatocellular carcinoma cells to sorafenib [15]. And in cervical cancer, high concentrations of oleanolic acid could promote the process of Ferroptosis by enhancing ROS content to achieve tumor killing effect [16]. In the present study, by analysing the degree of activation of oxidative stress pathway in liver injury, we found that there was excessive activation of oxidative stress in liver injury. And the oxidative damage caused by oxidative stress is one of the main causes of liver injury. In contrast, oleanolic acid, as an antioxidant drug, can act on HMOX1 protein to inhibit the process of oxidative stress and suppress the occurrence of Ferroptosis, which is beneficial to the regeneration of liver tissue.

As our study shows, oxidative stress and Ferroptosis occur during liver injury. And targeting oxidative stress and Ferroptosis is also the mechanism of action of several drugs. And HMOX1 as an important antioxidant stress enzyme is the main target of many drugs. Scutellarin (SCU), a major component of Scutellaria baicalensis, acts on the Nrf2/HMOX1 pathway to inhibit oxidative stress and thus protect against acute alcoholic liver injury [17]. Nocardone (NOOT) can provide protection against CCl4-induced oxidative stress and liver injury through modulation of the Nrf2/HMOX1 pathway [18]. Oxyberberine (OBB), a major intestinal metabolite of Phellodendron, can exert good hepatoprotective effects by activating the Nrf2/HMOX1 pathway in red blood cells [19]. Intestinal polysaccharide (EPP) can also exert hepatoprotective effects by activating HMOX1 and inhibiting oxidative stress in liver injury [20]. Lonicera japonica polyphenols (LCPs) can promote the expression of HMOX1, regulate the intestinal environment and further reduce liver injury by inhibiting oxidative stress-related pathways and altering the composition of the intestinal microbiota [21]. Acetaminophen (APAP) overdose is a common drug-related liver injury, and related studies have shown that both methane-rich saline (MRS) and salvianolic acid C (SAC) can reduce oxidative stress damage in liver-injured tissues by activating the Nrf2/HMOX1 pathway and achieve hepatoprotective effects [22, 23]. In summary a variety of drugs can inhibit oxidative stress in liver injury through activation of HMOX1 protein to achieve hepatoprotective effects. Also, the occurrence of oxidative stress resulting in elevated ROS can promote the occurrence of Ferroptosis in liver injury, which further suggests that drugs can inhibit the occurrence of Ferroptosis upon activation of HMOX1. In acetaminophen (APAP)-associated pharmacological liver injury, both kaempferol (KA) and astaxanthin (ASX) can act with HMOX1, leading to HMOX1 protein activation, which inhibits the oxidative stress process and further inhibits the occurrence of Ferroptosis [24, 25]. In the present study, we also found that oleanolic acid, as an antioxidant drug, has a similar mechanism of action to the above drugs. HMOX1 protein is a key target of oleanolic acid to inhibit liver injury, and oleanolic acid can inhibit oxidative stress injury and Ferroptosis in the liver injury pathway by promoting high expression of HMOX1.

Currently oleanolic acid is still used as a clinical drug mainly for the treatment of acute hepatitis-related conditions. Therefore, current studies have also focused on the role of oleanolic acid in liver injury. In our study, a large amount of data has also been applied to investigate the potential targets and mechanisms of action of oleanolic acid in liver injury. However, it is worth mentioning that most of the liver injuries caused by various liver diseases evolve into cirrhosis and further into liver cancer. There are still few studies on oleanolic acid and liver cancer, but studies have also shown that oleanolic acid can inhibit the malignant cell behaviour of liver cancer cells *in vitro* [26] and can be used as part of nanomedicines for the treatment of liver cancer [27, 28]. However, oleanolic acid, as an active ingredient of traditional Chinese medicine, has relatively mild effects, and there is still no clinical example of oleanolic acid treating liver cancer. Therefore, this study aims to apply the big data of liver cancer to predict the anticancer effect of oleanolic acid and provide a research basis for the application of oleanolic acid in clinical practice. Our study also found that oleanolic acid in liver cancer could promote the development of Ferroptosis in liver cancer by promoting HMOX1. This suggests that oleanolic acid may increase the fragility of liver cancer cells by promoting Ferroptosis, thus making it easier for other anti-tumour treatments to kill the tumour.

## MATERIALS AND METHODS

### Data sources and pre-processing

In this study, liver tissue-related RNA sequencing data of various liver injuries were collected from the GEO database, including 122 cases of viral infection liver injury (VLJ) (GSE83148), 106 cases of liver transplantation-related liver injury (TLJ) (GSE145780) and 109 cases of alcoholic liver disease-related liver injury (ALJ) (GSE94417). Non-alcoholic fatty liver disease-associated liver injury (FLJ) 202 cases (GSE213621). One hundred and ten cases of normal liver tissues from the GTEx database were also collected and used as normal controls. As all groups of liver injury were from different disease types, in order to maintain good biological variation, we did not perform a de-batching analysis and only used the limma package for in-sample de-batching.

### Gene difference analysis and enrichment analysis

For different types of liver injury, genetic differential analysis was performed with liver tissues in the GTEx database as the control group, and GSEA analysis was performed with GO and ReactomePA as the reference gene set, while GSVA analysis [29] was performed with KEGG as the reference pathway set to finally determine the genetic characteristics of various liver injuries. Gene intersection was performed for the differential genes of the four liver injuries to obtain the genes that were elevated and decreased during liver injury, and gene enrichment analysis was performed with GO and ReactomePA as the reference gene set.

### Acquisition of drug targets and key targets

The action targets of oleanolic acid were obtained from four Chinese medicine databases, including TCMSP, TCMID, SymMap and TCM-ID, and the gene functions of the targets were analysed. In this study, the above liver injury genes and oleanolic acid targets were used to obtain the targets of oleanolic acid action on liver injury, and PPI analysis was performed to obtain the related protein networks. Based on the oleanolic acid-related RNA-seq (GSE120311, with PLC-PRF-5 cells), the drug's ability to regulate the genes was initially obtained, and the main targets of oleanolic acid acting on liver injury and the related PPI network were further identified.

### Key pathway analysis and immune infiltration analysis

The set of oxidative stress-related pathways was obtained from the MSigDB database, and GSVA analysis was applied to determine the enrichment of oxidative stress pathways in various types of liver injury. The expression of Ferroptosis-related markers in liver injury and the relationship with key gene expression were further analyzed. Finally, the Cibersort algorithm [30] was used to predict the infiltration of immune cells in liver tissue from liver injury and to explore the correlation between immune cells and the main targets of oleanolic acid.

### Analysis of oleanolic acid targets associated with liver injury in cancer

This study first explored the expression, mutations and immune pathways of oleanolic acid targets associated with liver injury in cancer using TCGA pan-cancer data. Liver cancer related RNA-seq data were collected from three online databases, TCGA(TCGA-LIHC), GEO(GSE14520, GSE116174, GSE54236) and ICGC(ICGC-LIRI), for 968 cases of patients with liver cancer. A total of 340 patients with cirrhosis were also collected for comparison in GEO(GSE15654, GSE84044) and 479 cases from normal liver tissues were obtained from three online databases, TCGA(TCGA-LIHC), GEO(GSE14520, GSE54236) and GTEx. The RNA-seq data were transformed into FPKM data and normalized accordingly. ScRNA-seq data in GEO166635 was used to analyse the relationship between HMOX1 protein and Ferroptosis-related genes in the liver cancer microenvironment. GSE120311 was used as oleanolic acid-associated liver cancer cell RNA-seq data to observe the effect of oleanolic acid on relevant targets in liver cancer. In this study, 231 liver cancer patients with low expression of HMOX1 and high expression of HDAC1 were selected as Class I and 231 liver cancer patients with high expression of HMOX1 and low expression of HDAC1 were selected as Class II based on the median expression of HMOX1 and HDAC1. Gene difference analysis and GSEA enrichment analysis were performed on the data of the two groups of patients. Estimate analysis and Cibersort analysis were also performed to investigate the differences in immune microenvironment between the two groups of patients. Finally, the expression and methylation of HMOX1 in hepatocellular carcinoma patients were investigated using the ULCAN database. In this study, the oncopredict package was applied for drug sensitivity analysis. Immunotherapy sensitivity was predicted using TIDE [31] in 600 patients with liver cancer, and patients with higher TIDE scores were less sensitive to immunotherapy.

### Drug analysis and statistical analysis

A molecular docking approach was used to validate the interactions between oleanolic acid and key targets proteins. The software used in this study includes R Studio (R4.2.1), Cytoscape 3.7.2, Prism 9, Autodock4 and Pymol. Statistical results at p<0.05 were considered statistically significant.

### Availability of data and materials

The datasets analyzed in this work may be found in the Supplementary Materials or contact with the first author.

## Supplementary Material

Supplementary Table 1
