# Supplementary material for: Integrative transcriptome and single-cell sequencing technology analysis of the potential therapeutic benefits of oleanolic acid in liver injury and liver cancer
Source: Aging (Albany NY). 2023 Dec 20;15(24):15267–86. doi: 10.18632/aging.205349 (PMC10781501; doi:10.18632/aging.205349)
Supplement: Supplementary Table 1 [file aging-15-205349-s001.pdf]

SUPPLEMENTARY TABLE

Supplementary Table 1. The targets of oleanolic acid in Chinese medicine databases.

Gene_name
AMY2A
CCND1
CASP3
CASP8
CASP9
CYP1A2
CYP3A4
NQO1
HDAC1
HMGB1
HMOX1
ICAM1
MET
NFE2L2
PLA2G1B
POLB
PPARA
PTEN
PTGIR
PTGS1
PTGS2
PTPN1
PTPN2
TOP1
TOP2A
UGT2B10
NAMPT
UGT2B11
AKR1B10
GPBAR1
ESR1
DPP4
AR
NR3C1
CHEK1
CCNA2
DPEP1
ESR2
PIM1
MAPK14
CDK2
NOS2
GSK3B
PPARG
